# Leigh Syndrome: Spectrum of Molecular Defects and Clinical Features in Russia

**DOI:** 10.3390/ijms24021597

**Published:** 2023-01-13

**Authors:** Denis Kistol, Polina Tsygankova, Tatiana Krylova, Igor Bychkov, Yulia Itkis, Ekaterina Nikolaeva, Svetlana Mikhailova, Maria Sumina, Natalia Pechatnikova, Sergey Kurbatov, Fatima Bostanova, Ochir Migiaev, Ekaterina Zakharova

**Affiliations:** 1 Research Centre for Medical Genetics, 115522 Moscow, Russia; 2 Research and Clinical Institute of Pediatrics Named After Yuri Veltischev of the Pirogov Russian National Research Medical University of the Ministry of Health of the Russian Federation, 125412 Moscow, Russia; 3 Russian Children’s Clinical Hospital, 119571 Moscow, Russia; 4 State Healthcare Institution of Sverdlovsk Region “Clinical and Diagnostic Center “Mother’s and Child Health Protection”, 620067 Ekaterinburg, Russia; 5 Morozov Children’s City Clinical Hospital, 119049 Moscow, Russia; 6 Research Institute of Experimental Biology and Medicine, Voronezh State Medical University Named after N. N. Burdenko, 394036 Voronezh, Russia; 7 Medical Center “Zdorovii Rebenok”, 394077 Voronezh, Russia; 8 Gemotest Laboratory LLC., 123112 Moscow, Russia

**Keywords:** Leigh syndrome, mitochondrial diseases, mitochondrial DNA, *SURF1*

## Abstract

Leigh syndrome (LS), also known as infantile subacute necrotizing encephalopathy, is the most frequent mitochondrial disorder in children. Recently, more than 80 genes have been associated with LS, which greatly complicates the diagnosis. In this article, we present clinical and molecular findings of 219 patients with LS and give the detailed description of three cases with rare findings in nuclear genes *MORC2, NARS2* and *VPS13D*, demonstrating wide genetic heterogeneity of this mitochondrial disease. The most common cause of LS in Russian patients are pathogenic variants in the *SURF1* gene (44.3% of patients). The most frequent pathogenic variant is c.845_846delCT (66.0% of mutant alleles; 128/192), which is also widespread in Eastern Europe. Five main LS genes, *SURF1, SCO2, MT-ATP6, MT-ND5* and *PDHA1*, account for 70% of all LS cases in the Russian Federation. Using next generation sequencing (NGS) technique, we were able to detect pathogenic variants in other nuclear genes: *NDUFV1, NDUFS2, NDUFS8, NDUFAF5, NDUFAF6, NDUFA10, SUCLG1, GFM2, COX10, PMPCB, NARS2, PDHB* and *SLC19A3,* including two genes previously associated with Leigh-like phenotypes—*MORC2* and *VPS13D*. We found 49 previously undescribed nucleotide variants, including two deep intronic variants which affect splicing.

## 1. Introduction

Primary mitochondrial diseases (MD) are the most common group of inherited metabolic disorders and are among the most common forms of inherited neurological disorders. MD are associated with pathogenic variants in nuclear DNA (nDNA) and mitochondrial DNA (mtDNA) genes which encode both structural and functional mitochondrial proteins and biochemically are characterized by defects of oxidative phosphorylation (OXPHOS) [1]. Despite the fact that mutations in the genes responsible for MD are rarely found individually, the total prevalence of this group of diseases is estimated as 1.6 per 5000 worldwide [2]. The most common MD in children is Leigh syndrome [3].

Leigh syndrome (OMIM#256000; LS), also referred to as subacute necrotizing encephalo(myelo)pathy, was first described by the British psychiatrist and neuropathologist Denis Archibald Leigh in 1951 [4]. LS is a severe progressive neurodegenerative disease, typically manifesting in infancy or early childhood (between 3 and 12 months of age), often following an acute (viral) infection or other metabolic challenges (e.g., surgery, prolonged fasting) and often rapidly deteriorating. Initial signs may be nonspecific and include failure to thrive, persistent vomiting, hypotonia and developmental regress with loss of previously acquired skills. Due to damage to the nervous system, the clinical picture is dominated by neurodegenerative symptoms, such as psychomotor retardation or regression, hypotonia, spasticity, dystonia, seizures, infantile spasms, movement disorders (e.g., chorea, dystonia, tremor, hemiballism), ataxia, dysphagia, respiratory disorders (episodes of hyper- or apnea, stridor breathing), neuropathy and myopathy. Non-neurological manifestations include cardiac, renal, hepatological and hematological abnormalities. Hypertrichosis is a frequent feature in LS patients with pathogenic variants in the *SURF1* gene [5,6]. The most common characteristic findings of LS include bilateral, symmetric focal hyperintensities in the basal ganglia, thalamus, substantia nigra, nucleus ruber, brainstem, cerebellar white matter, cerebellar cortex, cerebral white matter, or spinal cord on T2-weighted magnetic resonance imaging (MRI) [7].

LS is a genetically heterogeneous disorder and to date, is associated with pathogenic variants in more than 75 genes. It is characterized by all types of inheritance: maternal (for mutations in mtDNA), autosomal-recessive, autosomal-dominant, or X-linked (for nuclear-encoded genes). These circumstances significantly complicate the molecular genetic diagnosis of LS. The advent of next-generation sequencing (NGS) technology has greatly improved diagnostic yield for these genetically heterogeneous conditions. NGS provided confirmation of emerging cases and brought up diagnosis in atypical presentations as late-onset cases, which turned LS into a heterogeneous syndrome with variable outcomes [8,9]. An innovative “multimix” approach combining genomics, transcriptomics, metabolomics and proteomics can be invaluable for pinpointing the molecular cause of a disease. This is a rapidly expanding area, including systematic profiling of DNA, RNA, metabolites and proteins in various tissues, as well as continuous improvement in methodology, statistical modeling and bioinformatics analysis, combined with expert supervision, which can be invaluable in the diagnosis of LS [10].

In this article, we present our own data on the clinical, radiological, molecular and biochemical findings of 219 patients with LS and describe three clinical cases of rare findings in nuclear genes *MORC2, NARS2* and *VPS13D* identified by whole exome or whole genome sequencing and RNA analysis.

## 2. Results

### 2.1. Demography

The studied cohort consists of 219 patients (105 males/114 females) from 219 unrelated families. The average age at diagnosis of 193/219 patients was 29 months (range: 0–289 months). The average age of disease onset in 98/219 patients was 14 months (range: 0–132 months). The majority of the patients’ samples were delivered from several main hospitals, including Research and Clinical Institute of Pediatrics Named After Yuri Veltischev, Russian Children’s Clinical Hospital, Morozov Children’s City Clinical Hospital. The cohort of patients was multinational with the majority being Russians (*n* = 147). Among other nationalities were Ukrainians (*n* = 16), Belarusians (*n* = 5), Azerbaijanis (*n* = 5), Kumyks (*n* = 2), Armenians (*n* = 1), Kyrgyz (*n* = 1), Gypsies (*n* = 1), Tajiks (*n* = 1), Turkmen (*n* = 1), Abaza (*n* = 1) and Bashkirs (*n* = 1). For 37 patients, nationality was not specified.

### 2.2. Molecular, Clinical, Radiological and Biochemical Findings in Patients with Nuclear Genes’ Variants

#### 2.2.1. The SURF1 Gene

In total, 97 unrelated patients with pathogenic variants in the *SURF1* gene were included in the study with medical data available for 32 patients. For 65 retrospective samples of patients with the *SURF1* gene mutations, only genotype data were available for analysis. For these retrospective patients, the diagnosis of LS was defined in referral children’s hospitals based on the presence of the biallelic pathogenic variants in the *SURF1* gene and consistent clinical presentations. Average age at onset was evaluated as 16 (27/97) months (range: 3–44 months). Initial symptoms appeared within the first year of life in 13 patients (40.6%), in the second year—14 patients (43.8%) and in the remaining 5 patients (15.6%), it was not possible to establish the definite age of manifestation. In 12 patients (37.5%), the initial symptoms were provoked by bacterial or viral infection; in the remaining 20 (62.5%), the first symptoms developed without any external triggers or they were not reported in the statement. A total of 32 patients manifested with developmental delay (18/32; 56.3%), abnormal motor findings (9/32; 28.1%), hypotonia/floppiness (9/32; 28.1%) and feeding difficulties (7/32; 21.9%). At least two or more initial symptoms were combined in 18 patients (56.3%).

The most common symptoms were delay or regress of psychomotor development and muscular hypotonia (29/32; 90.6%). In 16 cases (50.0%), cerebellar syndrome was observed, manifested by ataxia and tremor. In 25.0% of patients (8/32), extrapyramidal symptoms were detected in the form of dystonia, myoclonus and choreiform hyperkinesis. Pyramidal symptoms were observed in three patients (9.4%). Epileptic seizures and/or convulsions were observed in two patients (6.3%). The most common visual symptom was nystagmus (15/32; 46.9%), followed by strabismus (10/32; 31.3%), partial optic nerve atrophy (6/32; 18.9%) and ptosis (4/32; 12.5%). Hypertrichosis was detected in 21 (65.6%) patients.

Brain MRI results were available in 22 patients. In 77.3% of patients (17/22), symmetrical, well-defined hyperintense on T2-WI and FLAIR mode and hypointense in T1-WI foci in the basal ganglia were visualized, in 86.4% (19/22)—in the brain stem (medium brain, bridge, medulla oblongata), 40.9% (9/22)—in the cerebellum, in 18.2% (4/22)—the thalamus, spinal cord involvement was visualized in 8 patients (36.4%), leukodystrophy presented in 4 (18.2%).

Blood lactate levels were known for 17 patients. It was elevated in all cases (average—4.2 mM/L, range: 2.2–8.5 mM/L). In 17 patients, concentration of different organic acids in the urine were also elevated: fumaric acid (10/17; 58.8%), 2-hydroxyisobutyrate (7/17; 41.2%), 3-hydroxybutyrate (6/17; 35.3%), succinate and 2-oxoglutaric acid (5/17; 29.4%) and lactate (4/17; 23.5%). Other intermediate metabolites of the tricarboxylic acid cycle were significantly less common. In 5 patients (29.4%), the levels of organic acids were within the reference values. The measurement of amino acids and acylcarnitines by tandem mass spectrometry revealed an increased concentration of 3-hydroxybutyryl carnitine in 5 out of 21 patients (23.8%). At the same time, no changes were detected in 16 patients.

**Figure 1:**
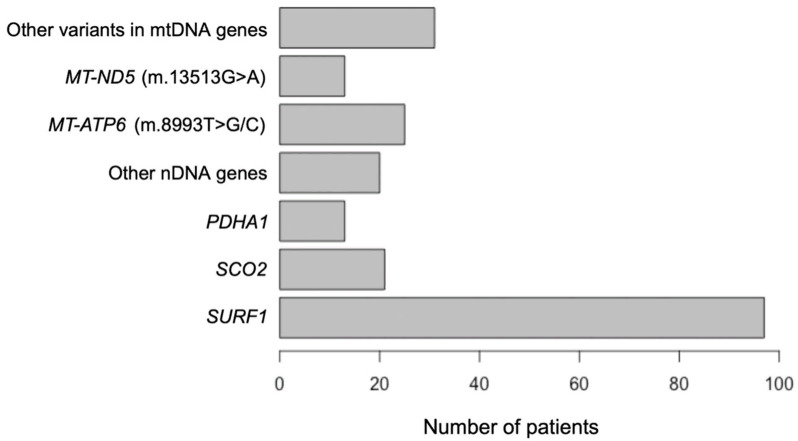
Spectrum and frequency of causative genes leading to Leigh syndrome in 219 patients from Russia.

**Figure 2:**
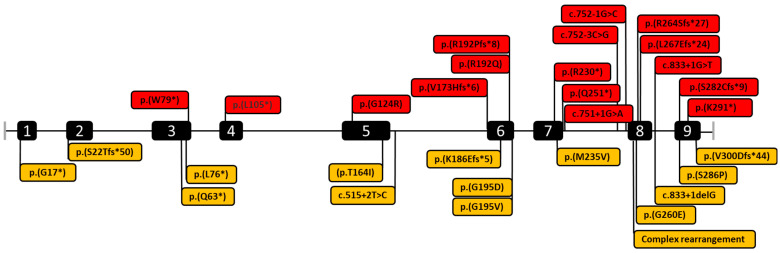
The *SURF1* gene variants revealed in this study. Red color—pathogenic variants described previously in the Human Gene Mutation Database; orange—novel variants identified in patients from this study.

**Figure 3:**
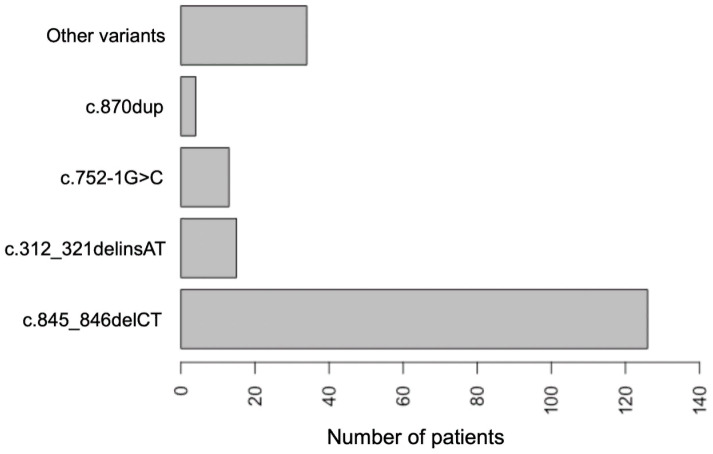
Spectrum and frequency of pathogenic variants in the *SURF1* gene in 97 patients.

Molecular genetic studies were performed in all 97 patients. Variants in the *SURF1* gene (OMIM* 185620; NM_003172.4) are frequent cause of LS in patients from Russia; this gene accounts for 44.3% of all cases (Figure 1). 32 different nucleotide variants were detected on 194 alleles from 97 unrelated families, including 14 new variants and 2 complex pathogenic variants (Figure 2). Among 32 variants: 8/32 (25.0%) were missense, 8/32 (25.0%) nonsense, 8/32 (25.0%) frameshift, 6/32 (18,8%) splicing and 2/32 (6.25%) complex rearrangements. The c.845_846delCT (p.Ser282CysfsTer9) was the most common variant with an allelic frequency of 66.0% (128/192); c.312_321delinsAT (p.Leu105Ter)—7.7% (15/194); c.752-1G>C—6.7% (13/194); c.870dup (p.Lys291Ter)—2.1% (4/194) and other variants—17.5% (34/194) (Figure 3). In 38 (39.2%) patients, the variant c.845_846delCT was detected in homozygous state and in 50 (51.5%) patients in compound heterozygous state.

#### 2.2.2. The *SCO2* Gene

A total of 21 unrelated patients with pathogenic variants in the *SCO2* gene were included in the study; medical data were available for 16 patients. Average age at onset was evaluated as 4 (16/21) months (range: 0–11 months). In 6 patients (37.5%), the initial symptoms were provoked by viral infection or vaccination and in the remaining 10 (62.5%), the first symptoms developed without any external triggers. Initial symptoms included delayed/regressed psychomotor development (6/16; 37.5%), hypotonia/floppiness (3/16; 18.8%), respiratory distress (3/16; 18.8%), feeding difficulties (2/16; 12.5%) and one case of cardiomyopathy (1/16; 6.3%).

In the clinical picture, respiratory symptoms (stridor, apnea) and cardiac pathology (hypertrophic cardiomyopathy, arrhythmia/conduction disturbances) come to the fore, accounting for 93.8% (15/16) and 68.8% (11/16) respectively. Hypotonia and dystonia were detected in 13 (81.3%) and 37.5% of patients (6/16) respectively. Among the extrapyramidal symptoms, it is worth noting the opisthotonos manifestation. Epileptic seizures and/or convulsions were observed in 5 patients (31.3%). Pyramidal symptoms were observed in 4 patients (25.0%). Ophthalmic manifestations included ptosis (5/16; 31.3%), nystagmus and strabismus (4/16; 25.0%) and partial atrophy of the optic nerve (1/16; 6.3%). The majority of patients had subfebrile fever of unclear etiology.

Brain MRI results were available in 15 patients. In 30.0% of patients (5/15), symmetrical, well-defined hyperintense on T2-WI and FLAIR mode and hypointense in T1-WI foci in the basal ganglia were visualized, in 13.3% (2/15)—in the brain stem (medium brain, bridge, medulla oblongata); leukodystrophy presented in 1 (6.7%). In 10 patients (66.7%), MRI of the brain revealed no characteristic changes.

Blood lactate level was elevated in 8 patients (average—5.7 mM/L, range: 3.3–9.1 mM/L). In 10 patients, the concentration of urine organic acids was also elevated: 3-hydroxybutyrate (7/10; 70.0%), 2-hydroxyisobutyrate and lactate (6/10; 60.0%), fumaric acid and 2-oxoglutaric acid (4/10; 40.0%), succinate (3/10; 36.4%). Increased concentration of other intermediate metabolites of the tricarboxylic acid cycle were significantly less common. In 1 patient (8.3%), the levels of organic acids were within the reference values. In all patients (16/21), the concentration of amino acids and acylcarnitines in blood were in the reference ranges.

Pathogenic variants in the *SCO2* gene (OMIM*604272; NM_005138.3) were found in 9.6% of Leigh syndrome cases in the Russian Federation (Figure 1). A total of 7 different nucleotide variants were detected on 42 alleles from 21 unrelated families, including 4 new variants c.202G>A (p.Gly68Arg), c.227_230del (p.Leu76ProfsTer2), c.533C>T (p.Ala178Val) and c.763C>T (p.Arg255Trp). Among 7 variants, 5/7 (71.4%) were missense and 2/7 (28.6%) frameshift variants. The c.418G>A (p.Glu140Lys) was the most common variant with an allelic frequency of 83.3% (35/42); c.16_17insAGCATGCAGCAGTGACTCA (p.Arg6GlnfsTer82)—4.8% (2/42); other variants—11.9% (5/42). In 14 (63.6%) patients, the variant c.418G>A was detected in the homozygous state, in 8 (36.4%) patients in the compound heterozygous state.

#### 2.2.3. The PDHA1 Gene

A total of 13 unrelated patients with pathogenic variants in the *PDHA1* gene were included in the study; medical data were available for 10 patients. Average age at onset was evaluated as 6 (8/10) months (range: 0–18 months). In 2 patients (20.0%), the initial symptoms were provoked by viral infection or vaccination, in the remaining 8 (80.0%), the first symptoms developed without any external triggers. Initial symptoms included hypotonia/floppiness (6/10; 60.0%) and delayed/regressed psychomotor development (3/10; 30.0%). In one patient, hypotonia was combined with respiratory disorders and in two with ptosis.

The most common symptom was muscle hypotonia/weakness (9/10; 90.0%). In 70.0% of cases (7/10), cerebellar symptoms in the form of ataxia were observed. In 6 patients (60.0%), a delay in psychomotor development was revealed. In addition, 10.0% of patients (1/10) presented with dystonia and extrapyramidal symptoms. Pyramidal symptoms and epileptic seizures and/or convulsions were observed in 2 patients (20.0%). Moreover, 2 patients (20.0%) clinically diagnosed with polyneuropathy. Ophthalmic manifestations included ptosis (4/10; 40.0%), nystagmus and strabismus (1/10; 10.0% and 2/10; 20.0% respectively) and partial atrophy of the optic nerve (1/10; 10.0%).

Brain MRI results were available in 9 patients. In 66.7% of patients (6/9), symmetrical, well-defined hyperintense on T2-WI and FLAIR mode and hypointense in T1-WI foci in the basal ganglia were visualized. In one patient, changes were detected in the brainstem and thalamus (1/9; 11.1%), in another—an isolated lesion of the cerebellum (1/9; 11.1%). In 2 patients (22.2%), MRI of the brain revealed no characteristic changes.

Blood lactate levels were known for 9 patients. It was elevated in all cases (average—6.5 mM/L, range: 3.0–14.0 mM/L). In 5 patients, different organic acids in the urine were also elevated: 2-hydroxyisobutyrate, 3-hydroxybutyrate and lactate (5/5; 100.0%), pyruvate (4/5; 80.0%), 2-oxoglutaric and methylmalonic acids (2/5; 40.0%). The study of amino acids and acylcarnitines by tandem mass spectrometry revealed an increase in alanine in 4 out of 6 patients (66.7%).

Pathogenic variants in the *PDHA1* (OMIM*300502; NM_000284.4) gene were found in 5.9% of LS cases in the Russian Federation (Figure 1). A total of 8 different nucleotide variants were detected on 13 alleles from 13 unrelated families, including 2 new variants (c.1158_1159insCAGTGGATCAAGTTTA (p.Lys387GlnfsTer50), c.1102_1103insTCTACT (p.Tyr369_Ser370insPheTyr). Among 8 variants: 6/8 (75.0%) were missense and 2/8 (25.0%) frameshift variants. The c.787C>G (p.Arg263Gly) was the most common variant with an allelic frequency of 30.7% (4/13); c.1132C>T (p.Arg378Cys)—23.1% (3/13); other variants—46.2% (6/13).

### 2.3. Molecular, Clinical, Radiological and Biochemical Findings in Patients with mtDNA Genes’ Variatns

In total, 68 unrelated patients with pathogenic variants in mtDNA were included in the study; medical data were available for 37 patients. Average age at onset was evaluated as 20 (33/37) months (range: 0–132 months). Initial symptoms appeared within the first year of life in 18 patients (48.6%), in the second year—14 patients (36.8%) and in the remaining 5 patients (13.2%) it was not possible to establish the definite age of manifestation. In 5 patients (13.2%), the initial symptoms were provoked by bacterial/viral infection or vaccinations, in 2 patients (5.3%) after head injury, in the remaining 31 (81.6%), the first symptoms developed without any external triggers or they were not reported in the statement. The initial symptoms were developmental delay (12/38; 31.6%), cerebellar symptoms (10/38; 26.3%), hypotension/lethargy (7/38; 18.4%) and convulsive seizures (6/38; 15.8%). At least two or more initial symptoms were observed in 18 patients (56.3%). Ophthalmological changes were the initial symptoms in 5 patients (5/38; 13.2%).

The most common symptom was muscular hypotonia (24/37; 64.9%). Pyramidal symptoms were observed in 20 patients (54.0%). Epileptic seizures and/or convulsions were observed in 12 patients (32.4%). In 43.2% of patients (16/37), extrapyramidal symptoms were detected in the form of dystonia, myoclonus and choreiform hyperkinesis. In 11 cases (29.7%) cerebellar syndrome was observed, manifested by ataxia. The most common visual symptom was strabismus (14/37; 37.8%), followed by partial optic nerve atrophy and ptosis (5/37; 13.5%) and nystagmus (3/37; 8.1%). Hypertrichosis was detected in 3 (8.1%) patients with m.13513G>A (2/3) and m.8344A>G (1/3) variants.

Brain MRI results were available in 33 patients. In 75.8% of patients (25/33), symmetrical, well-defined hyperintense on T2-WI and FLAIR mode and hypointense in T1-WI foci in the basal ganglia were visualized, in 39.4% (13/33)—in the brain stem (medium brain, bridge, medulla oblongata); in 21.2% (7/33)—the thalamus; 3.0% (1/33)—in the cerebellum, leukodystrophy presented in 3 (9.1%).

Blood lactate levels were known for 24 patients. It was elevated in all cases (average—5.7 mM/L, range: 2.8–11.8 mM/L). In 21 patients, different concentration of organic acids in the urine was also elevated: 2-hydroxyisobutyrate and fumaric acid (12/21; 57.1%), 3-hydroxybutyrate (11/21; 52.4%), succinate (10/21; 47.6%), 2-oxoglutaric acid (9/21; 42.9%), lactate (7/21; 33.3%) and pyruvate (4/21; 19.1%). Other intermediate metabolites of the tricarboxylic acid cycle were significantly less common. In 7 patients (33.3%), the levels of organic acids were within the reference values. Tandem mass spectrometry revealed high concentrations of alanine (4/18; 22.2%); propionylcarnitine, 3-hydroxy-isovaleryl-/2-methyl-3-hydroxy-butyrylcarnitine (2/18; 11.1%). Two patients with m.8993T>G mutation (11.2%) showed low citrulline concentration. In 9 patients (50.0%), concentration of urine organic acids and amino acids and acylcarnitines in the blood was within the reference ranges.

Together, pathogenic variants in mtDNA (NC_012920.1) constitute the second most common cause of LS in the Russian Federation (31.1%; 68/219) (Figure 1). The m.8993T>C/G variant in the *MT-ATP6* gene (OMIM*516060) was the most common (25/68; 36.8%), followed by the m.13513G>A (13/68; 19.1%) in the *MT-ND5* gene (OMIM*516005). Other variants (m.13094T>C, m.14441T>C, m.3945C>A, m.8839G>C, m.8344A>G, m.3697G>A, m.10191T>C, m.10197G>A, m.14487T>C, m.14459G>A) account for 44.1% (30/68). Homoplasmic pathogenic variants in blood samples were observed in 35 patients (51.5%). In the remaining patients, the level of heteroplasmy in blood ranged from 22% to 90%. In 16/21 blood samples and in 5/8 urine sediment samples, mothers were heteroplasmic carriers of the pathogenic variants.

### 2.4. Clinical Cases

**Figure 4:**
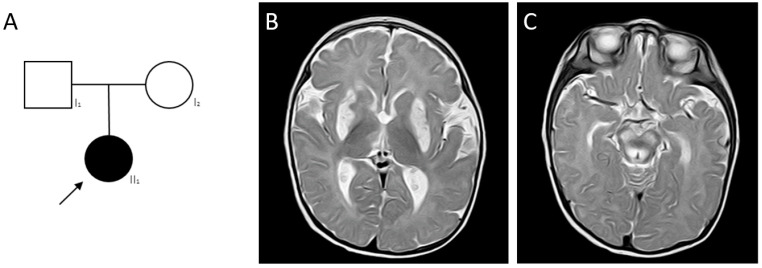
Patient 1 with LS caused by pathogenic variants in the *MORC2* gene. (**A**)—Pedigree of patient 1. MRI images of the brain; (**B**)—bilateral of hyperintensity on T2W mode in the putamen; (**C**)—changes in the pontine tegmentum.

Patient 1, girl, 7 m. old and the only child in the family (Figure 4A). At the age of 7 months, she was admitted to an emergency department with seizures, the “swordsman” posture and series seizures (up to 2–3 series per day). Patient showed a psychomotor development delay—no rolling over, no crawling and no sitting. The disease manifested at 4 m. with psychomotor regress and seizure attacks.

At the time of examination, the patient’s neurological status: muscle weakness, seizures, hypotonia, tetraparesis and pseudobulbar syndrome. Cerebral, meningeal symptoms were not revealed. The examination demonstrated no gaze fixation and convergent strabismus. The muscle tone and tendon reflexes from the hands and legs were reduced, D = S. The girl was lying in the “frog” pose. Pathological reflexes were not present.

The following stigmas of dysembryogenesis have been identified: microcephaly, small forehead, asymmetric cranium, hypertrichosis, long bright eyelashes and thick eyebrows.

MS/MS revealed an increased concentration of alanine (Ala) and tetradecanoyl-carnitine (C14:2), a decreased concentration of glycine (Gly) and tyrosine (Tyr). Blood lactate was significantly elevated—7.7 mM/L.

On a series of brain MRI images, a symmetrical hyperintense signal on T2W and FLAIR in the basal ganglia, thalamus, hypothalamus abnormality, delayed myelination, lesions in mesencephalon, pedunculus cerebelli and moderate ventriculomegaly (Figure 4B,C) were revealed.

Spinal muscular atrophy was excluded on the first line of the diagnosis. WES analysis revealed previously described pathogenic variant c.79G>A (p.Gly27Lys) in the *MORC2* (OMIM*616661; NM_001303256.3) gene in heterozygous state [11]. Segregation analysis confirmed the de novo state of the variant in the proband.

The second family with c.79G>A (p.Gly27Lys) *MORC2* variant with Leigh-like phenotype is also included in our series.

**Figure 5:**
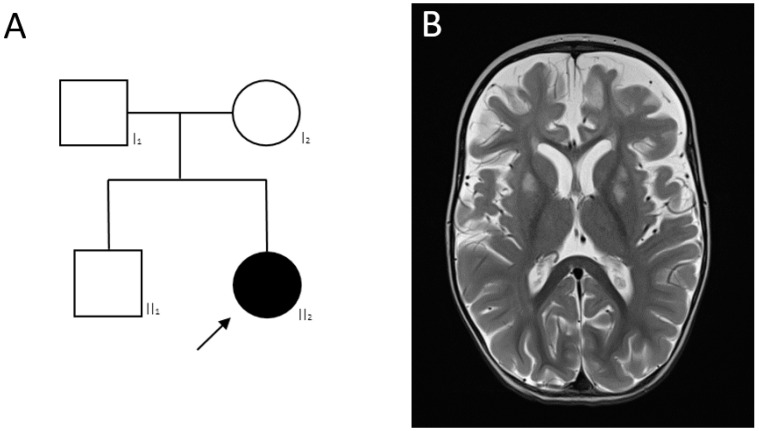
Patient 2 with LS caused by pathogenic variants in the *NARS2* gene. (**A**)—Pedigree of patient 2. MRI images of the brain; (**B**)—Bilateral of hyperintensity on T2W mode in the putamen and globus pallidus and atrophy in the frontal region.

Patient 2, girl, 10 m. old. The second child in this family, the elder sibling is 6 years old, healthy (Figure 5A). At the age of 3.5 months, she was consulted with delayed psychomotor and pre-speech development, dystonic muscle tone, with elements of hypertension in the distal extremities, hypotension in the adductor muscles of the neck, oculomotor disorders (horizontal nystagmus).

Brain MRI showed a symmetrical hyperintensive signal in T2W and Flair in the basal ganglia in the projections of the putamen and globus pallidus, as well as atrophy in the frontal region (Figure 5B).

EEG—there is no epileptic activity.

EMG: myopathy: SMA, PNP excluded.

Urine organic acids analysis showed increased concentration of 2-hydroxyisobutyrate, 3-hydroxybutyrate, suberic acid. MS/MS detected an increase in the concentration of alanine. The level of serum lactate was also significantly elevated—5.5 mM/L.

On examination at the age of 8 months the girl presented hypomimic, with discoordinated movements of the eyeballs. Muscle tone was dystonic (crossed): hypertonicity of the upper extremities, D > S, lower extremity tone D < S, no head control. Later, at the age of 10 months, epilepsy joined.

**Figure 6:**
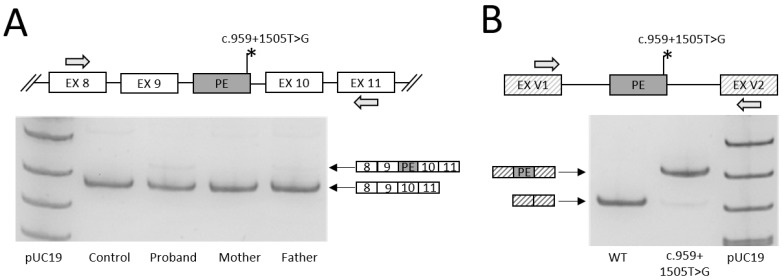
The results of mRNA analysis for the c.959+1505T>G variant. (**A**)—Analysis of mRNA from patient’s fibroblasts. Visualization of PCR products by PAGE. (**B**)—Results of the minigene assay. Visualization of plasmid-specific PCR products by PAGE. PE—pseudoexon, EX V1 and EX V2—constitutively spliced exons of the pSpl3-Flu2-mTK vector, gray arrows—location of PCR primers. pUC19—DNA ladder.

WGS analysis revealed two variants in the *NARS2* gene (OMIM*612803; NM_024678.6): large 9.6 kb deletion comprising exons 8 and 9 in compound heterozygous state with the c.959+1505T>G variant located in the intron 9 and predicted to affect splicing. None of the variants have been described previously. Using Sanger sequencing, we validated the deletion, which is NC_000011.9:g.78187758_78197406del, in heterozygous state in proband and her mother. To determine the effect of the c.959+1505T>G variant on the gene’s splicing, we analyzed the *NARS2* mRNA isolated from the patient’s cultured fibroblasts. The *NARS2* cDNA fragment was amplified with primers located in 8 and 11 exons to eliminate the mRNA isoforms from the deletion-containing allele. Visualization of PCR products on polyacrylamide gel electrophoresis (PAGE) revealed an additional low intensity band in patient’s and father’s samples (Figure 6A). The band was extracted, reamplified and Sanger sequenced. We revealed that the band represents an insertion of the 41 bp pseudoexon located in the intron 9 and activated by the c.959+1505T>G variant, which creates the strong donor splice site. The insertion r.959_960ins41 leads to frameshift and the formation of the premature stop codon in exon 10 (p.Ile321TrpfsTer12), which could explain the low intensity of the PCR product, as the corresponding isoforms are substrates of nonsense mediated mRNA decay (NMD). To analyze the effect of c.959+1505T>G in the absence of NMD we performed minigene assay. The fragments of the *NARS2* intron 9 with and without c.959+1505T>G were cloned between two constitutively spliced exons of the pSpl3-Flu2-mTK vector and transfected into HEK293T cells. After 48 h, the mRNA was extracted and reverse transcribed. The visualization of minigene-specific PCR products demonstrated the similar splicing pattern with insertion of 41 bp in the vast majority of mRNA isoforms. Thus, according to the ACMG criteria (PS3, PM2, PM3 supportive), the intronic variant c.959+1505T>G should be classified as likely pathogenic.

**Figure 7:**
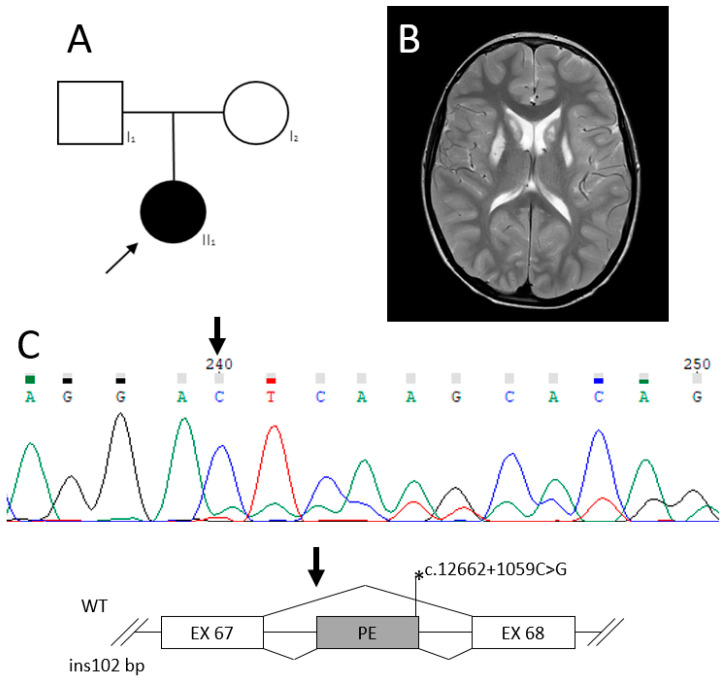
Patient 3 with LS caused by pathogenic variants in the *VPS13D* gene. (**A**)—Pedigree of patient 3. MRI images of the brain; (**B**)—bilateral of hyperintensity on T2W mode in the putamen and globus pallidus and atrophy in the frontal region; (**C**)—Sanger sequencing of cDNA fragment with c.12662+1059C>G variant and the scheme of splicing pattern for WT and mutant types. The lower peaks correspond to transcript containing 102 bp. insertion. The bold arrow on the chromatogram and on the scheme below indicates the beginning of the pseudoexon insertion.

Patient 3, a boy, 6 y.o., single child from non-consanguineous parents (Figure 7A). The child was born in term, at the 38th week from the 1st pregnancy, with intrauterine development delay and placental insufficiency. Apgar 8/9. Early development with delay: independent walk from 1.5 years with atactic gait. At 3 years of age, the boy gained 3 words to speak and at 6 years—20 words. Karyotyping was normal. Angelman syndrome was also excluded.

At the age of 2 years, epileptic seizures began, with loss of consciousness lasting up to 15 min, with a frequency of 1 time in 1–2 months. EEG showed no epileptic activity. The patient was treated with two antiepileptic drugs, with effect. At the age of 3 years, brain MRI showed symmetrical hyperintensive signal on T2W in basal ganglia (putamen and caudate nuclei) (Figure 7B). Laboratory tests revealed high blood lactate concentration—3.35 mM/L (*n* ≤ 2.2 mM/L) with normal amino acids and acylcarnitines in blood and normal organic acids in urine.

On examination at 6 years of age: head circumference—50 cm (−1.33 SD), height—114 cm (−0.49 SD), weight—18 kg (−1.07 SD). Some phenotype abnormalities such as low physical development, strabismus, long philtrum, macrostomy, dysplastic ears, blond hair, thin lips and epicanthus were noted. In neurological status: diffuse muscular hypotonia with high knee and Achilles reflexes, atactic gait on a wide base, mostly walks on tiptoes.

Based on the combination of clinical data and the results of MRI studies, the diagnosis of Leigh syndrome was assumed. WES analysis revealed variants in the *VPS13D* gene (OMIM*608877; NM_015378.4): heterozygous variant c.8687C>T (p.Thr2896Met) which was not described earlier and a heterozygous deep-intronic variant c.12662+1059C>G. The segregation analysis showed that the missense variant was inherited from the father and the intron variant was inherited from the mother.

The effect of the variant c.12662+1059C>G in the *VPS13D* gene on splicing was analyzed in patient’s blood RNA sample. It was found that the deep intronic variant c.12662+1059C>G at the mRNA level leads to the inclusion of a pseudoexon and the insertion of 102 bp r.12662_12663ins102 (Figure 7C), which leads to a frameshift and the formation of a premature stop codon in pseudoexon (p.Arg4221_Thr4222insLysThrTyrTer). According to the ACMG criteria (PS3, PM2, PM3 supportive), the variant c.12662+1059C>G should be classified as likely pathogenic. The missense variant c.8687C>T (p.Thr2896Met) according to the AMG criteria was defined as uncertain significance variant.

## 3. Discussion

We present data on the clinical, biochemical and genetic heterogeneity of a cohort of 219 patients with a genetically confirmed diagnosis of Leigh syndrome. Our data will help understand the clinical and genetic features of Leigh syndrome, especially in the Russian population. These data are useful for early diagnosis and differential diagnosis with other mitochondrial, neuromuscular and neurometabolic diseases.

Analyzing the clinical features of the entire population that participated in our study we found that, regardless of the molecular cause, the most common and initial symptoms of the disease are psychomotor retardation or regression of skills and muscle hypotonia/weakness. At the same time, there are differences in the age of manifestation: the average age of manifestation in pathogenic variants in the *SCO2* gene is 4 months, while for variants in mtDNA, manifestation is typical at a later age—20 months. Typically, the age of onset in patients with the *SURF1* gene mutations ranges from 3 to 6 months [5], but in our series, the average age of the manifestation is 16 months. The major clinical presentation of *SURF1*-associated Leigh syndrome is cerebellar signs such as ataxia (40.6%). Ataxia is also a common symptom in *PDHA1* variants (70.0%). Pyramidal symptoms in the form of spastic paraplegia/tetraparesis are more typical for patients with mtDNA variants (54.0%) than the *SURF1* gene cases (9.4%). Although spasticity in *SURF1* appears in the later period of the disease, in cases of mtDNA variants, it was observed at early stages in some patients. Epilepsy, as another neurological sign, was observed in 31.6% (12/38) of patients with mtDNA variants and only in 9.4% (2/32) of the *SURF1* gene patients. This is consistent with the literature data, since epilepsy is not a characteristic symptom for *SURF1* patients, while it is common for mtDNA mutations [12].

Cardiological abnormalities are not typical for LS although hypertrophic cardiomyopathy is one of the frequent symptoms in patients with *SCO2* variants. Hypertrophic cardiomyopathy, profound hypotonia along with stridor and other respiratory problems could be the leading symptoms for these patients. From our series, 10/16 patients presented with early fatal form of *SCO2*-deficiency with cardiomyopathy [13]. The presence of a febrile syndrome not associated with infectious factors and characterized by a constant or episodic increase in body temperature from subfebrile to febrile is frequently observed in the *SCO2* patients. The absence of characteristic LS changes on MRI in some of the cases can be explained by the early onset, severe and rapid course of the disease which is also suspected in the literature [14]. Interestingly, febrile syndrome has been described as a separate phenotypic trait for patients with a confirmed mutation and the *SCO2* gene [15]. In patients with m.13513G>A mutation, cardiac pathology with Wolff–Parkinson–White syndrome is typical and well described in the literature [16].

The LS associated with pathogenic variants in mtDNA is characterized by an extraordinary clinical polymorphism, depending on the type of the variant and the level of heteroplasmy. In severe cases, clinical signs may manifest during the first two years, including the early neonatal period, while a lighter phenotype may develop in a childhood. Although in our series, early onset and severe neurological symptoms were noted in the patients with the m.8993T>C/G variant with high mutant load (>90%) for the m.13513G>A variant, we did not reveal strict genotype–phenotype correlations—among 12 patients with LS, the heteroplasmy in blood cells was 40–100% while in 5 patients with isolated LHON phenotype (not included in the study [17]), heteroplasmy overlapped strongly with LS patients (24–60%).

A distinctive feature of *SURF1*-associated LS is the presence of hypertrichosis, which was detected in 65.6% (21/32) of patients in our series. Wedatilake et al. in their observation report the presence of hypertrichosis in 41.0% of patients, which is slightly lower than our data [18]. According to the literature, hypertrichosis is a characteristic sign and, in the presence of developmental delay or other nonspecific neurological symptoms, can hint a diagnosis [19]. Dysmorphic symptoms were identified in some of our patients (wide forehead, blond hair, wide bridge of the nose, thin lips, hypertelorism, epicanthus). The main characteristics of clinical, neuroradiological and biochemical data for main molecular causes of LS are shown in Table 1.

The general pattern on brain MRI includes symmetrical lesions in the basal ganglia. These changes are associated with paralysis of the brain stem, cerebellum and thalamus. The involvement of the brain stem for the *SURF1* gene patients is highly prevalent—86.4% (19/22). Among mtDNA cases, almost 73.5% of patients (25/34) have changes in the basal ganglia. In 7 patients, leukodystrophy was detected which is consistent with previously published data [20].

A biochemical abnormality common to all patients is an elevated blood lactate level. The maximum mean value of 11 mM/L was observed in the patient with m.8993T>C/G variant in the *MT-ATP6* gene. Changes in the spectrum of organic acids are relatively non-specific and include high concentrations of the Krebs cycle metabolites: 2-hydroxyisobutyrate, 3-hydroxybutyrate, 2-oxoglutaric acid, lactate, pyruvate, fumarate, succinate, etc. In cases with *PDHA1*-associated LS, a high level of alanine in blood is frequently observed and for the m.8993T>C/G variant low citrulline in blood (2/10; 20%) was noted which was previously reported in the literature [21].

According to E. Fassone and S. Rahman, the most frequently observed biochemical abnormality in LS is an isolated deficiency of complex I, which accounts for 34% of cases [22], whereas complex IV deficiency is the cause of approximately 15% of LS cases worldwide [23]. However, in Russia, more than half of the patients have isolated complex IV deficiency (54.5%) and complex I deficiency ranks only second (21.5%). The most common cause of Leigh syndrome in Russian patients is pathogenic variants in the *SURF1* gene (44.3%). The most frequent variant is c.845_846delCT (66.0%), followed by c.312_321delinsAT (7.7%). Variants c.845_846delCT and c.312_312delinsAT are thought to be the most common variants in Eastern European population. Pronicki et al., in a series of 21 Polish patients, showed the presence of the c.845_846delCT variant in all patients [24]. However, according to Sofou et al. variants in the *SURF1* gene are not so frequent—8/130 patients [25]. Furthermore, a common cause of LS in Chinese patients is the *ECHS1* gene mutations, with a frequent variant c.463G>A (p.Gly155Ser) [26], which indicates a potential founder effect already described for several mutations in LS [27].

Among 16 novel identified variants, there are 6 missense substitutions c.491C>T (p.Thr164Ile), c.584G>A (p.Gly195Asp), c.856T>C (p.Ser286Pro), p.584G>T (p.Gly195Val), c.779G>A (p.Gly260Glu), c.703A>G (p.Met235Val), 3 nonsense variants c.49G>T (p.Gly17Ter), c.187C>T (p.Gln63Ter), c.227T>A (p.Leu76Ter), 3 frameshift c.554_555insA (p.Lys186GlufsTer5), c.65del (p.Ser22ThrfsTer50), c.899_902del (p.Val300AspfsTer44), 2 splicing variants (c.515+2T>C, c.833+1del) and 2 complex rearrangements.

Compound heterozygotes for frequent deletion c.845_846delCT and missense variant c.584G>A (p.Gly195Asp), as well as splicing variant c.752-1G>C and missense variant c.491C>T (p.Thr164Ile) have a milder phenotype compared to homozygous c. 845_846delCT. Both patients manifested later (14 m.) and are alive at 10 years of age. Assumptions that the missense variants can lead to a milder course have been established before [28,29], but there are also publications not confirming this observation [30,31].

Using massive parallel sequencing we were able to detect pathogenic variants in other 16 LS nuclear genes: *NDUFV1, NDUFS2, NDUFS8, NDUFAF5, NDUFAF6, NDUFA10, SUCLG1, GFM2, COX10, PMPCB, NARS2, PDHB, SLC19A3* and *IARS2*, including two genes previously associated with Leigh-like phenotypes—*MORC2* and *VPS13D* [11,32]. A total of 23 unique novel variants were found, two of which are located deep in the intron region and affect splicing.

## 4. Materials and Methods

### 4.1. Editorial Policies and Ethical Considerations

This work was carried out in accordance with The Code of Ethics of the World Medical Association Declaration of Helsinki for experiments involving humans. The study was approved by the local ethics committee of the Federal State Budgetary Institution “Research Center for Medical Genetics”. Informed consent was obtained from all patients.

### 4.2. Patients

We recruited 219 patients (114 female and 105 male) with defined molecular diagnosis of LS analyzed in the laboratory of inherited metabolic diseases between 2005 and 2022. The inclusion criteria were progressive neurological deterioration with evidence of brainstem and/or basal ganglia involvement, motor delay, high levels of serum lactate and/or the presence of pathogenic/likely pathogenic variants in causative genes. Clinical, biochemical, neuroradiological and molecular data were recorded from the patient’s medical documents where available.

Diagnosis of LS was confirmed with different methods—176 patients were revealed by screening for frequent mutations in the *SURF1, SCO2* and *PDHA1* genes and mtDNA point mutations or full gene sequencing, 26 patients were revealed on targeted genes panels, 15 on WES, 2 on WGS. For two patients with *VPS13D* and *NARS2* gene variants, the whole genome sequencing was performed with TruSeq DNA PCR-Free sample preparation kit on NovaSeq 6000 (Illumina, San Diego, CA, USA).

### 4.3. DNA and RNA Extraction, Analysis and Sanger Sequencing

Genomic DNA was extracted from blood samples with the use of QiaAMP DNA-mini kit (Qiagen, USA), following the manufacturer’s protocol.

Total RNA was isolated from whole blood cells and fibroblasts using Total RNA Purification Plus Kit (Norgene, Thorold, ON, Canada). The first strand of cDNA was synthesized using ImProm-II™ Reverse Transcriptase (Promega, Madison, WI, USA) and oligo(dT) primers.

Detection of frequent mutations was carried out by single-stranded conformation polymorphism (SSCP) analysis or multiplex ligation-dependent probe amplification (MLPA) with subsequent validation of the identified changes according to Sanger.

Sanger sequencing was performed using an ABI Prism 3500XL (Thermo Fisher Scientific, Waltham, MA USA), following the manufacturer’s protocol. Genetic variants are named according to the GRCh37 (hg19) genome assembly.

### 4.4. Metabolite Analyses

MS/MS analysis of acylcarnitines in dried blood spots was performed using “NeoGram Amino Acids and Acylcarnitines TandemMass Spectrometry Kit” (Perkin Elmer, Helsinki, Finland). The urinary organic acids were extracted by diethyl ether/ethyl acetate, derivatized and analyzed by GC/MS 7890A/5975C (Agilent Technologies, USA) withHP-5MS [33].

### 4.5. WES and WGS

Whole genome sequencing was performed with TruSeq DNA PCR-Free sample preparation kit on NovaSeq 6000 (Illumina, San Diego, CA, USA).

Bioinformatic pipeline: sequence reads were aligned to human reference genome GRCh37 (hg19) using Burrows–Wheeler Aligner (http://bio-bwa.sourceforge.net/ (accessed on 1 September 2022)). Single-nucleotide variants and small insertions and deletions (indels) were called with Strelka2 Small Variant Caller (https://github.com/Illumina/strelka (accessed on 1 September 2022)) and the Genome Analysis Toolkit v.4 (https://gatk.broadinstitute.org/ (accessed on 1 September 2022)). Structural variants were identified with Manta (https://github.com/Illumina/manta (accessed on 1 September 2022)). The reported variants were annotated with their genomic coordinates, allele frequency (gnomAD database, http://gnomad.broadinstitute.org/ (accessed on 1 September 2022)), functional consequence and impact level on the gene product using SnpEff v5 (http://pcingola.github.io/SnpEff/ (accessed on 1 September 2022)). Variants were prioritized by the consensus score of the set of bioinformatic tools, which predict the pathogenicity of the variant and the deleterious effect on protein (SIFT, SIFT4G, Polyphen2, MutationAssessor, FATHMM, PROVEAN, DEOGEN2, LRT, PrimateAI, MetaSVM, MetaLR, SpliceAI, MMsplice, SPiP, Spidex). Data analysis was performed with in-home NGSData-Genome interface.

All variants revealed by massive parallel sequencing were validated by the Sanger sequencing in all patients and both their parents where possible.

Variants were named according to the HGVS nomenclature and validated using VariantValidator (https://variantvalidator.org/ (accessed on 1 September 2022)). The novel variants were classified according to ACMG recommendations [34].

### 4.6. Minigene Assay

The 357 bp fragment of the *NARS2* intron 9 from patient (NC_000011.9:g.78187868_78188224) was cloned between two constitutively spliced exons (V1 and V2) of the pSpl3-Flu2-mTK vector [https://doi.org/10.1038/s41525-022-00315-y]. Plasmids with minigenes (250 ng plasmids in 24-well plate cell) were transfected into HEK293T cells (ATCC CRL-3216™) using Lipofectamine 3000 reagent (Thermo Fisher Scientific, Waltham, MA, USA). After 48 h, cells were harvested for RNA isolation and reverse transcription. Minigene-specific primers with 6-FAM modification located in the exons V1 and V2 were used to amplify the splicing products, which were further visualized by polyacrylamide gel electrophoresis (PAGE) with subsequent gel purification (if needed) and Sanger sequencing.

## 5. Conclusions

Leigh syndrome is a clinical and genetically heterogeneous disease that manifests in childhood. Analysis of clinical, biochemical, neuroradiological and molecular genetics data in a large series of 219 patients with LS is supposed to improve the understanding of the clinical characteristics and diagnostic algorithms of this group of mitochondrial diseases in the Russian Federation. Analysis of clinical data did not allow to differentiate patients within a group of LS by the molecular defect. Although *SURF1*, *SCO2* and some *PDHA1*-patients could be clinically well-defined, collection of such symptoms as median disease manifestation at 13 months, muscular hypotonia, hypertrichosis, ataxia and bilateral lesions in brainstem and/or basal ganglia are typical for the *SURF1* gene cases. Median manifestation at 3 months, stridor, opisthotonos and hyperthermia in combination with profound muscular hypotonia (SMA-like phenotype) are specific for *SCO2*-patients. Median manifestation at 4 months, muscular hypotonia and highly elevated lactate and pyruvate in blood and urine organic acids (>100-fold increase) allow to suspect PDH-complex defect. Clinical pictures of the patients with pathogenic variants in other LS genes are more heterogenous, overlapping and unspecific, so massive parallel sequencing techniques are justified and highly effective to carry out the diagnosis in these patients. Leveraging of frequent population-specific mutation tests in the first line of laboratory diagnostics could speed up confirmation of the diagnosis and help to avoid unnecessary studies.

Rare cases of LS could be caused by genes not directly involved in OXPHOS function—*MORC2*-associated SMA-like phenotypes and *VPS13D*-associated ataxia + phenotypes are a bright illustration of LS phenocopy. We clearly showed that whole genome sequencing could improve the diagnostic yield as a second pathogenic allele could represent a large structural defect or deep intronic nucleotide variant.

## Figures and Tables

**Table 1:** Main clinical symptoms in Leigh syndrome patients with frequent pathogenic variants.

Symptoms	*SURF1*	*SCO2*	*PDHA1*	m.8993T>G/C	m.13513G>A	Other mtDNA
Onset (median age, range; months)	13.0 (3–44)	3.0 (0–11)	4.0 (0–18)	4.5 (0.5–24)	15.0 (0–132)	14.5 (3–60)
Delay or regress of psychomotor development	90.6% (29/32)	50.0% (8/16)	60.0% (6/10)	72.7% (8/11)	70.0% (7/10)	87.5% (14/16)
Muscle hypotonia/weakness	90.6% (29/32)	75.0% (12/16)	90.0% (9/10)	81.8% (9/11)	50.0% (5/10)	62.5% (10/16)
Ataxia	40.6% (13/32)	0.0% (0/16)	70.0% (7/10)	18.2% (2/11)	30.0% (3/10)	37.5% (6/16)
Epileptic seizures and/or convulsions	6.3% (2/32)	31.3% (5/16)	20.0% (2/10)	36.4% (4/11)	30.0% (3/10)	31.3% (5/16)
Pyramidal symptoms	9.4% (3/32)	25.0% (4/16)	20.0% (2/10)	36.4% (4/11)	80.0% (8/10)	50.0% (8/16)
Dystonia	18.8% (6/32)	43.8% (7/16)	10.0% (1/10)	27.3% (3/11)	30.0% (3/10)	56.3% (9/16)
Myoclonus and choreiform hyperkinesis	18.8% (6/32)	6.3% (1/16)	10.0% (1/10)	36.4% (4/11)	0.0% (0/10)	31.3% (5/16)
Hypertrichosis	65.6% (21/32)	0.0% (0/16)	0.0% (0/10)	0.0% (0/11)	20.0% (2/10)	6.3% (1/16)
Nystagmus	46.9% (15/32)	25.0% (4/16)	10.0% (1/10)	18.2% (2/11)	30.0% (3/10)	25.0% (4/16)
Strabismus	31.3% (10/32)	25.0% (4/16)	20.0% (2/10)	18.2% (2/11)	60.0% (6/10)	37.5% (6/16)
Ptosis	12.5% (4/32)	31.3% (5/16)	40.0% (4/10)	9.1% (1/11)	30.0% (3/10)	6.3% (1/16)
Optic nerve atrophy	18.9% (6/32)	6.3% (1/16)	10.0% (1/10)	9.1% (1/11)	20.0% (2/10)	12.5% (2/16)
Feeding difficulties	25.0% (8/32)	18.8% (3/16)	20.0% (2/10)	9.1% (1/11)	10.0% (1/10)	12.5% (2/16)
Dysmorphic features	18.8% (6/32)	25.0% (4/16)	20.0% (2/10)	0.0% (0/11)	40.0% (4/10)	0.0% (0/16)
Liver damage	21.9% (7/32)	0.0% (0/16)	20.0% (2/10)	18.2% (2/11)	40.0% (4/10)	6.3% (1/16)
Cardiological symptoms	21.9% (7/32)	68.8% (11/16)	30.0% (3/10)	18.2% (2/11)	70.0% (7/10)	18.8% (3/16)
Hematological changes	6.3% (2/32)	37.5% (6/16)	10.0% (1/10)	18.2% (2/11)	20.0% (2/10)	0.0% (0/16)
Respiratory problems	9.4% (3/32)	93.8% (15/16)	10.0% (1/10)	63.6% (7/11)	10.0% (1/10)	12.5% (2/16)
Lesions on brain MRI:						
Basal ganglia	77.3% (17/22)	30.0% (5/15)	66.7% (6/9)	77.8% (7/9)	66.7% (6/9)	85.7% (12/14)
Brain stem	86.4% (19/22)	13.3% (2/15)	11.1% (1/9)	44.5% (4/9)	33.3% (3/9)	35.7% (5/14)
Thalamus	18.2% (4/22)	0.0% (0/15)	11.1% (1/9)	11.1% (1/9)	22.2% (2/9)	28.6% (4/14)
Cerebellar	40.9% (9/22)	0.0% (0/15)	11.1% (1/9)	11.1% (1/9)	0.0% (0/9)	0.0% (0/14)
Spinal cord	36.4% (8/22)	0.0% (0/15)	0.0% (0/9)	0.0% (0/9)	0.0% (0/9)	0.0% (0/14)
Leukodystrophy	18.2% (4/22)	6.7% (1/15)	0.0% (0/9)	11.1% (1/9)	11.1% (1/9)	7.1% (1/14)
Lactate level (median concentration, range; mM/L)	3.9 (2.2–8.5)	4.9 (3.3–9.1)	6.1 (3.0–14.0)	5.5 (1.2–16.0)	5.5 (3.6–10.5)	4.4 (4.1–7.0)

## Data Availability

Not applicable.
